# Overcoming hypoxia-induced resistance of pancreatic and lung tumor cells by disrupting the PERK-NRF2-HIF-axis

**DOI:** 10.1038/s41419-020-03319-7

**Published:** 2021-01-13

**Authors:** Alina Küper, Jennifer Baumann, Kirsten Göpelt, Melanie Baumann, Christopher Sänger, Eric Metzen, Philip Kranz, Ulf Brockmeier

**Affiliations:** Institut für Physiologie, Universität Duisburg-Essen, 45147 Essen, Germany

**Keywords:** Cancer, Cell biology

## Abstract

Hypoxia-induced resistance of tumor cells to therapeutic treatment is an unresolved limitation due to poor vascular accessibility and protective cell adaptations provided by a network, including PERK, NRF2, and HIF signaling. All three pathways have been shown to influence each other, but a detailed picture remains elusive. To explore this crosstalk in the context of tumor therapy, we generated human cancer cell lines of pancreatic and lung origin carrying an inducible shRNA against NRF2 and PERK. We report that PERK-related phosphorylation of NRF2 is only critical in Keap1 wildtype cells to escape its degradation, but shows no direct effect on nuclear import or transcriptional activity of NRF2. We could further show that NRF2 is paramount for proliferation, ROS elimination, and radioprotection under constant hypoxia (1% O_2_), but is dispensable under normoxic conditions or after reoxygenation. Depletion of NRF2 does not affect apoptosis, cell cycle progression and proliferation factors AKT and c-Myc, but eliminates cellular HIF-1α signaling. Co-IP experiments revealed a protein interaction between NRF2 and HIF-1α and strongly suggest NRF2 as one of the cellular key factor for the HIF pathway. Together these data provide new insights on the complex role of the PERK-NRF2-HIF-axis for cancer growth.

## Introduction

Hypoxically induced resistance to chemo- or radiotherapy is still an unresolved issue in tumor treatment. This is mainly due to lower formation of oxygen radicals in hypoxic tumor areas, due to poor bio-accessibility of drugs at the site of action and due to cell-protective mechanisms of cancer cells and their tumor mircoenvironment. For adaptation in this harsh environment of deprived oxygen and nutrients, dividing cancer cells can activate various cellular pathways (e.g., HIF, KRAS, PI3K, WNT, PERK, MYC, NRF2) to ensure proliferation and survival. Unfortunately, targeting a single pathway usually promotes the selection for bypass mechanisms that lead to tumor resistance by either mutations in the drug target or activation of alternative signaling^1^. A promising strategy to avoid resistance might be a simultaneous blockade of several pathways that are upregulated in cancer. Since the NRF2- antioxidant response was shown to be important for tumor cells^2,3^, our goal was to examine its role in the context of the PERK-NRF2-HIF-axis to understand the underlying pathway interactions and to evaluate its benefit as a cancer target.

The nuclear transcription factor NF-E2-related factor 2 (NRF2) signaling provides an efficient cellular mechanism to restore oxidative homeostasis. Upon excessive ROS production, NRF2 gets stabilized, forms heterodimers with small MAF proteins in the nucleus and binds the regulatory antioxidant response element (ARE) to induce expression of a whole set of phase II and III enzymes (e.g., glutathione S-transferases (GSTs), NAD(P)H:quinone oxidoreductase 1 (NQO1), superoxide dismutase (SOD), multiple drug transporter (MDR1-3)), and thus ensures the detoxification of the cells^4^. Its major repressor is the Kelch-like ECH-associated protein 1 (Keap1), an adaptor molecule for the cullin 3 ubiquitin ligase complex that provides disposal of NRF2^5^. The cysteine-rich Keap1 protein also functions as a ROS sensor: oxidation of several highly reactive cysteines interrupts the weak NRF2-Keap1 interaction and leads to the accumulation of NRF2^6^. As a major cytoprotective factor, NRF2 prevents cancer initiation and progression in normal cells but also supports growth and chemoresistance in tumor cells^7,8^.

Another cellular stress pathway, the unfolded protein response (UPR), gets activated upon ER stress caused by misfolded proteins, hypoxia or ROS and aims to nullify protein aggregation and to regain ER balance. One of the three sensors of the UPR is the PKR-like endoplasmic reticulum kinase (PERK)^9^. Notably, besides phosphorylation of its main target eIF2α, PERK was also shown to phosphorylate NRF2, which supports its dissociation from Keap1^10^. Accumulating evidence points to a key role of PERK in tumor progression and metastasis^11^, and its prosurvival activity via ATF4 and NRF2 makes it an attractive cancer target^12,13^.

Apart from PERK, NRF2 also interacts with the HIF pathway^14^. HIF signaling is the classical response to oxygen deprivation. Thereby, the hypoxia-induced factor HIF gets stabilized and adapts metabolism, vascularization and inhibits apoptotic factors to ensure cell survival and thus correlates with chemo- and radioresistance of solid tumors. Recent data demonstrated that NRF2 targets a functional ARE upstream of *HIF-1α*^15^, which triggers the HIF response. However, if there is an additional interaction between NRF2 and HIF-1*α* on the protein level is still unclear.

In this study, we investigated the interplay between NRF2, PERK, and HIF-1*α* in a pancreatic and a lung cancer cell line to increase the understanding of this cellular network for clinical applications. We were able to demonstrate that cancer cell growth was inhibited efficiently by depletion of NRF2 solely under constant hypoxia. We could also confirm that targeting NRF2 further upstream at its activator PERK is only rational if the cancer cell line carries a wildtype *Keap1* gene. Finally, we identified NRF2 as an essential factor for the HIF pathway at least partially through protein interaction with HIF-1α.

## Results

### NRF2 requires its phosphorylation by PERK only to avoid Keap1-dependent degradation

**Fig. 1:**
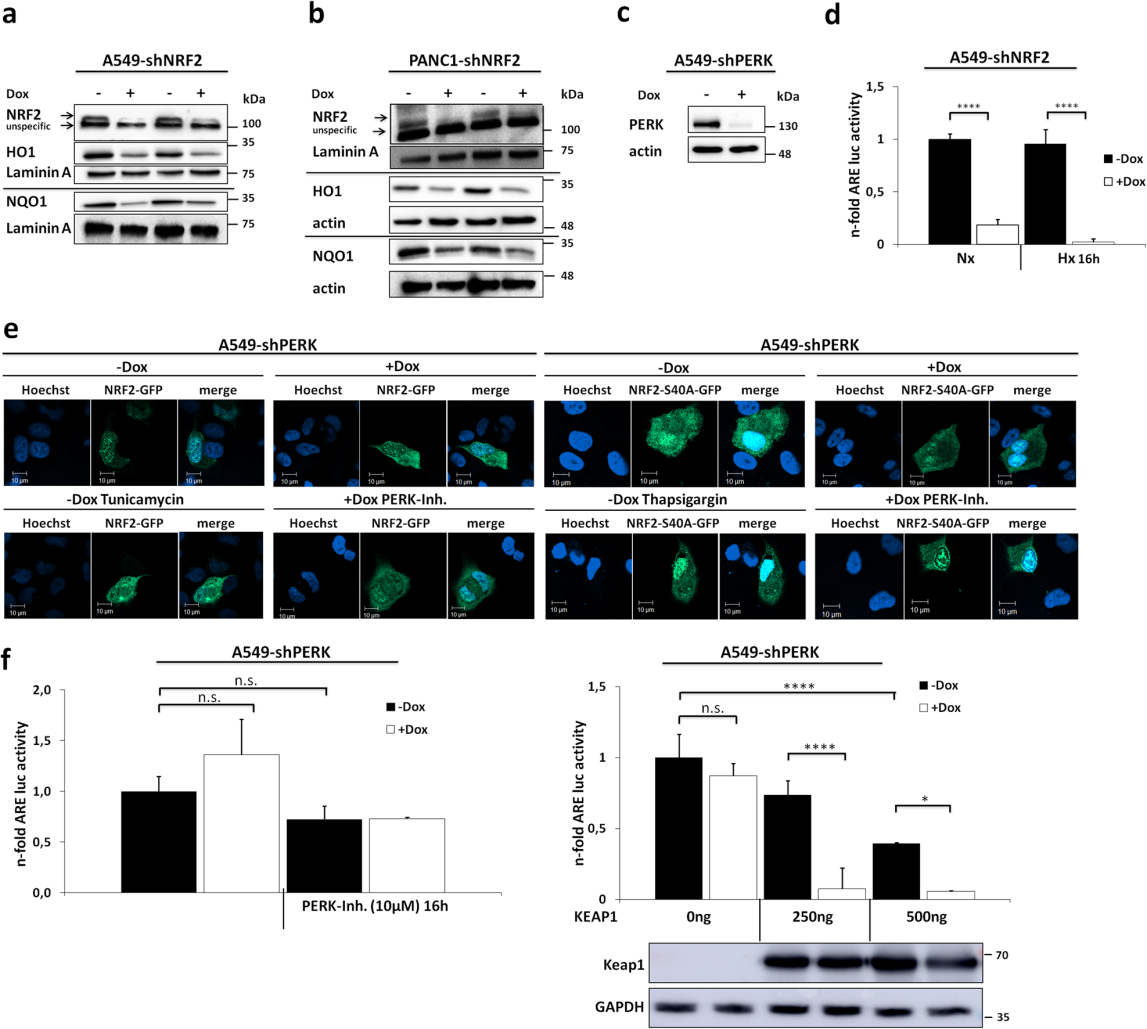
Relevance of PERK for nuclear translocation, transcriptional activation, and stability of NRF2 in A549-shPERK cells. **a**–**c** Western blot analysis of total lysates of lentiviral-transduced A549-shNRF2, PANK1-shNRF2, and A549-shPERK cells showing protein expression for NRF2, its downstream targets HO1 and NQO1 (**a**, **b**) and PERK (**c**) 48 h after KD induction. Laminin A and actin were detected as loading controls. **d** Luciferase activity assay was performed in A549-shNRF2 cells transfected with pGL3-8xARE, expressing firefly luciferase downstream of an antioxidant-responsive element (ARE), and pGL4.74 (renilla luciferase) for normalization. Seventy-two hours after KD induction and 16 h in normoxia or hypoxia, whole-cell lysates were used for measurement. **e** Confocal microscopy of A549-shPERK cells transfected with pEGFP-NRF2 (left panel) or pEGFP-NRF2-S40A (right panel). UPR was stimulated either by 1 µM Thapsigargin or 1 µM Tunicamycin. In addition to a PERK KD, 10 µM PERK-Inhibitor GSK2656157 (PERK-Inh.) was used to eliminate any PERK signaling. Hoechst 33342 was used to stain cell nuclei. **f** To test transcriptional activity of NRF2 without PERK, cells were transfected with pGL3-8xARE and pGL4.74. Seventy-two hours after PERK KD and 24 h after chemical PERK inhibition, luciferase assays were performed. In addition, KEAP1 dysfunction in A549 cells was counterbalanced by co-transfection with different amounts of plasmid Flag-Keap1 expressing functional KEAP1 protein (right panel). Western blot analysis of total lysates with a Flag antibody showing protein expression for Flag-Keap1. GAPDH was detected as loading control.

To examine the role of PERK for NRF2 signaling, we generated the cell lines A549-shNRF2, PANC1-shNRF2, and A549-shPERK carrying a doxycycline-inducible knockdown (KD) system for NRF2 and PERK. We confirmed an almost complete target KD for all three cell lines on protein level via western blot (Fig. 1a–c). We further examined the NRF2 downstream targets hemoxygenase 1 (HO1) and NQO1 after KD and found a decrease for both proteins as well (Fig. 1a, b). The interruption of the NRF2 signal pathway via NRF2 KD in A549 cells was also validated by a luciferase-based gene reporter assay measuring NRF2 transcriptional activity under normoxia and oxygen deprivation (Fig. 1d). PERK-dependent phosphorylation of NRF2 was described as essential for its nuclear translocation and transcriptional activation in mouse fibroblasts^10^. Here, we were able to test this observation in the cancer cell line A549-shPERK that shows high levels of cellular NRF2 due to a dysfunctional Keap1 protein. Initially, we overexpressed plasmid-encoded GFP-tagged wildtype NRF2 (NRF2-GFP) and detected its cellular localization (Fig. 1e, left panel): under non-stressed conditions in the presence of PERK (-Dox), NRF2 was located in the cytoplasm and the nucleus. However, neither activation of PERK with Tunicamycin nor depletion of PERK alone or combined with chemical inhibition (+Dox, PERK Inh.) did affect its nuclear levels. To exclude a compensational phosphorylation of NRF2 by other kinases such as protein kinase C or casein kinase 2 as suggested in former studies^16,17^, the experiment was repeated with a non-phoshorylatable NRF2 mutated at amino acid position 40 from Ser to Ala (S40A). However, even the NRF2-S40A-GFP protein showed unchanged nuclear localization (Fig. 1e, right panel). These results point to a nuclear translocation of NRF2 that is independent from PERK and from its phosphorylation status at S40. To assess if PERK is required for NRF2 functionality, a luciferase reporter assay was performed in A549-shPERK cells to measure NRF2 transcriptional activity (Fig. 1f, left panel). Similar to its translocation, NRF2 activity was unaffected by the KD of PERK. Finally we intended to examine the role of PERK in cancer cells with a functional Keap1 protein as a NRF2-repressor. Therefore, we overexpressed plasmid-encoded wildtype Keap1 to suppress endogenous dysfunctional Keap1 in A549-shPERK cells and checked for NRF2 activity. Indeed, increasing amounts of functional KEAP1 not only led to a dose-dependent loss of NRF2 activity, but also exposed a strong dependency of NRF2 function for PERK (Fig. 1f, right panel). This experiment confirmed that NRF2 phosphorylation by PERK is mandatory to allow NRF2 escape from Keap1-dependent degradation.

### NRF2 promotes survival of cancer cells under continuous hypoxia

**Fig. 2:**
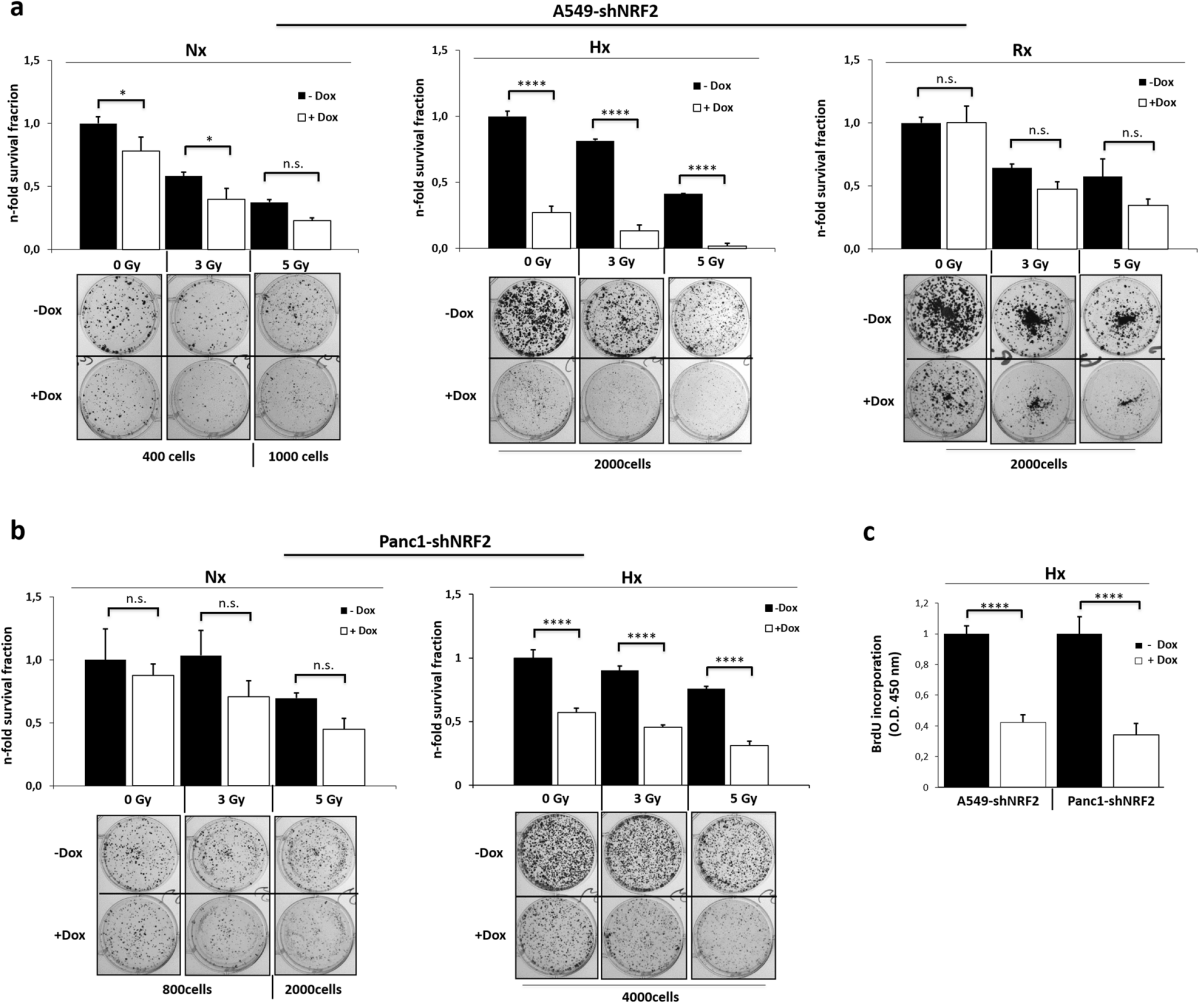
Survival of irradiated A549 and PANC1 cells after NRF2 KD depends on the oxygen level. Clonogenic survival assay of A549-shNRF2 cells (**a**) and PANC1-shNRF2 cells (**b**) after irradiation combined with NRF2 KD. Hypoxic A549-shNRF2 cells were reoxygenated (RX) right after radiation treatment and kept in normoxia until the end of the experiment. **c** BrdU proliferation assay in hypoxia was performed 72 h after NRF2 KD.

Next, we assessed the role of NRF2 for survival of irradiated cancer cells by performing clonogenic survival assays (CFAs) (Fig. 2). After NRF2 depletion and irradiation alone, we observed a dose-dependent decrease in colony formation ability for both cell lines (compare Fig. 2a, b). Under hypoxic condition we noticed a drastic reduction in cell growth compared to the normoxic samples when NRF2 was depleted. It is a well-established observation that hypoxic tumor areas show radioresistance during treatment due to decreased generation of reactive oxygen species^18^. Therefore, it was surprising that the combined treatment of NRF2 KD and irradiation under hypoxia maximized the detrimental effect for cell survival in both cell lines. In addition, we conducted CFAs under post-hypoxia and post-irradiation reoxygenation (RX) in A549-shNRF2 cells (Fig. 2a). Unexpectedly, the reoxygenation conditions abrogated the severe growth inhibition observed in hypoxia after NRF2 depletion. In addition, we determined cell proliferation under oxygen deprivation by the measurement of newly synthesized DNA using the thymidine analog BrdU. As shown in Fig. 2c, the depletion of NRF2 resulted in a strong growth inhibition in both cell lines.

### Influence of NRF2 on apoptosis, proliferation factors, cell cycle, and intracellular ROS

**Fig. 3:**
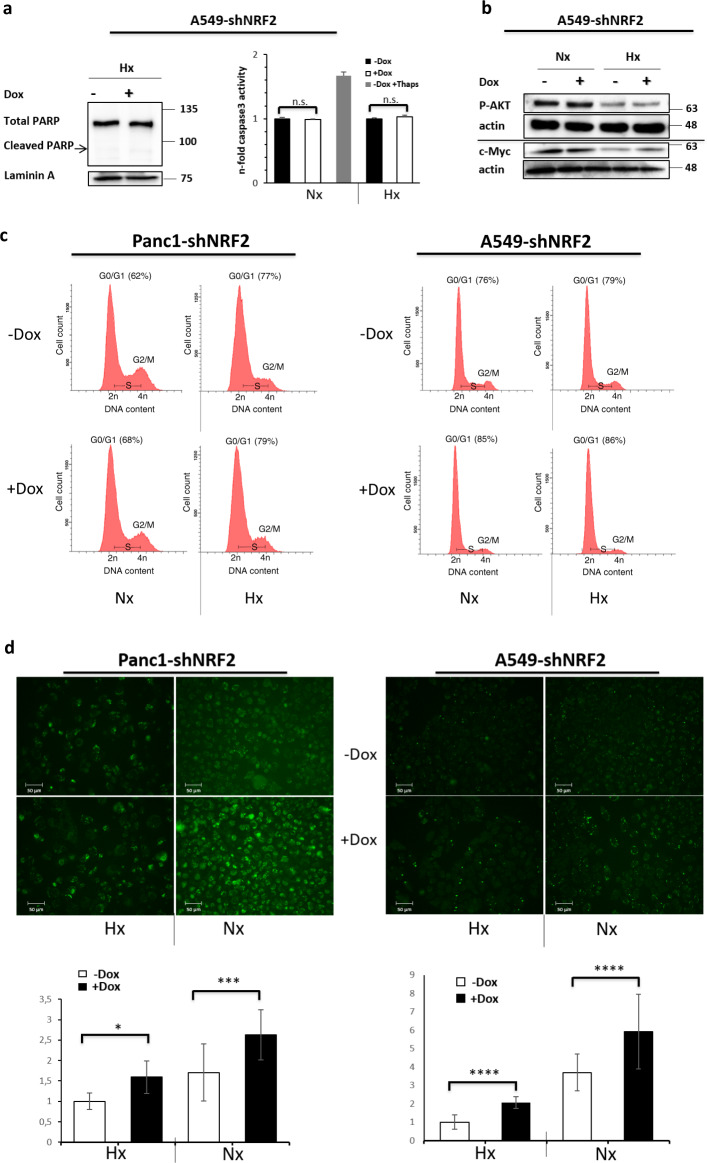
Effect of NRF2 deficiency on apoptosis, proliferation factors, cell cycle, and intracellular ROS. **a** NRF2 depletion does not trigger apoptosis. 48 h after NRF2 KD induction and 16 h of hypoxia, total cell lysates were subjected to western blot analysis for PARP with Laminin A as loading control (left panel) further caspase3 activity assay was analyzed (right panel). **b** Proliferation factors AKT and c-Myc are not affected by KD of NRF2. Whole-cell lysates from normoxic and hypoxic samples were prepared 72 h following NRF2 KD induction. Western blot analysis compared phospho-AKT (S473) and c-Myc protein levels, actin served as a loading control. **c** Cell cycle progression is independent of NRF2. PANC1-shNRF2 (left panel) and A549-shNRF2 cells (right panel) were incubated for 96 h after NRF2 KD under normoxic or hypoxic conditions before nuclei were stained with PI and analyzed for DNA content by FACS. **d** NRF2 counteracts oxidative stress independent of the oxygen level. PANC1-shNRF2 (left panel) and A549-shNRF2 cells (right panel) were incubated as under **c**. After 96 h, CellROX reagent (5 µM) was added to the culture medium for 2 h in order to visualize oxidative stress. The upper panels display representative fluorescence images. For each condition, 12 randomly chosen areas were photographed with the same parameters and the fluorescence signals were analyzed with IMAGEJ software (lower panels).

To delineate the detrimental effects after NRF2 depletion under hypoxia, we evaluated apoptosis in A549-shNRF2 cells, but no PARP cleavage or caspase3 activity was detectable (Fig. 3a). Next, we looked into the proliferation factors AKT and c-Myc on protein level, but besides a downregulation of both factors in hypoxia, no differences were observed when NRF2 was absent (Fig. 3b). Cell cycle progression was tested in PANC1-shNRF2 and A549-shNRF2 cells by FACS analysis but only revealed a marginal increase in the G0/G1 phase after NRF2 KD independent of the oxygen level (Fig. 3c). This effect could hardly explain the drastic growth phenotype in the CFAs. Accordingly, we further examined oxidative stress levels in the cells after depletion of NRF2. We used the intracellular ROS detection reagent CellROX, that once oxidized, emits a stable green fluorescence signal and is, therefore, suitable for visual quantification (Fig. 3d). As expected, we measured more intense ROS levels in normoxia than in hypoxia for both cell lines. However, more strikingly, we found increased ROS levels after NRF2 KD, which confirms a critical function of NRF2 in the antioxidant response.

### HIF-1α activity is strictly NRF2 dependent

**Fig. 4:**
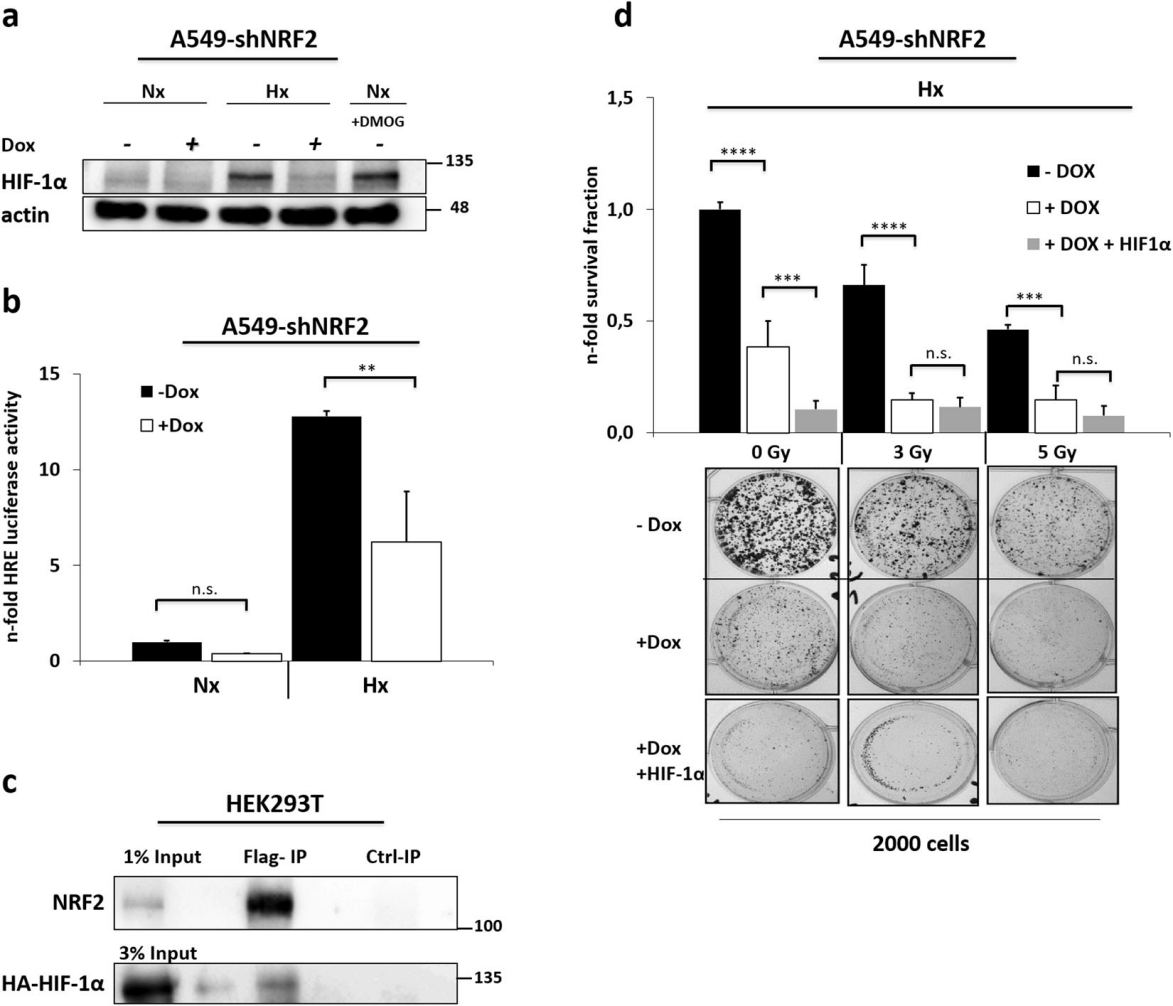
NRF2 can interact with HIF-1α and is essential for its activity. **a** After NRF2 KD for 48h and 16h in hypoxia, protein expression of HIF-1α and loading control actin was monitored by western blotting. HIF stabilizing compound DMOG (1 mM) was used as a positive control for HIF-1α in normoxia. **b** To detect transcriptional activity of HIF, a luciferase activity assay was performed in A549-shNRF2 cells transfected with pGLHIF1.3^43^, expressing firefly luciferase downstream of three hypoxia-responsive elements (3x HRE), and pGL4.74 (renilla luciferase) for normalization. Seventy-two hours after KD induction and 16 h in normoxia or hypoxia, whole-cell lysates were used for measurement. **c** IP experiment to detect protein interaction of NRF2 and HIF-1α. Plasmid Flag-NRF2 was co-transfected with vector p(HA)HIF1alpha (P402A,P564A), expressing a non-degradable/oxygen-stable form of HIF-1α, into HEK293T cells. NRF2 was pulled with an anti-Flag antibody (Flag-IP) and complex formation with HIF-1α was determined by western blot analysis using anti-NRF2 and anti-HA (HIF-1α) antibodies. 1–3% of total cell lysate was loaded as input. **d** Clonogenic survival assay in A549-shNRF2 cells under hypoxic conditions after irradiation combined with HIF-1α overexpression to compensate for KD of NRF2. Cells were transfected 24 h after KD induction with vector p(HA)HIF1α-P402A-P564A.

Considering that our NRF2 KD results showed growth effects exclusively under hypoxic conditions, we next focused on assessing the impact of NRF2 on hypoxia inducible factor HIF-1α by immunoblot analysis. As expected, oxygen-related degradation of HIF-1α was inhibited under hypoxia or after treatment with HIF stabilizing reagent dimethyloxaloylglycine (DMOG), (Fig. 4a). However, we noticed that the HIF-1α protein was completely missing in hypoxia when NRF2 was absent. In addition, we tested HIF transcriptional activity in a luciferase assay after NRF2 KD and observed a reduced HIF signal of about 50%, which supports our western blot findings (Fig. 4b). This raised the question if NRF2 could be a stabilizing binding partner for HIF-1α on the protein level. Indeed, CO-IP experiments revealed a reliable protein interaction between both transcription factors (Fig. 4c). Altogether the dependency of HIF-1α on NRF2 implied the possibility that the absence of HIF-1α could have caused the severe proliferation inhibition that we observed in our hypoxic growth assays (see Fig. 2). To address this question, CFAs were performed in hypoxia with a NRF2 KD and simultaneous plasmid-encoded HIF-1α expression. However, instead of a reversed growth inhibition by the continuous presence of HIF-1α we noticed an even stronger reduction in cell growth indicating that the transfection procedure and/ or HIF-1α overexpression might have a detrimental effect on the cells (Fig. 4d).

## Discussion

Over the last 2 decades, a set of kinases were found as upstream regulators of the NRF2 signaling pathway. Among others, protein kinase C (PKC)^16^, casein kinase (CK2)^17^, glycogen synthase kinase 3β (GSK-3 β)^19^ and PERK^10^ were all able to phosphorylate NRF2, but varied considerably regarding its cellular impact. For PERK, the exact phosphorylation site of NRF2 was not identified yet although it was stated otherwise^4^. It was also unclear whether the PERK-dependent phosphorylation of NRF2 residues only influences its degradation by Keap1 or directly affects its nuclear import or transcriptional activity^6^. Here, we demonstrated that neither nuclear translocation nor function of NRF2 relies on PERK activity in cancer cells. Admittedly, we cannot rule out that other kinases can compensate for PERK. However even so, this would still signifies that PERK is not essential for NRF2 activity. Further, the nuclear abundance of a S40 phosphorylation deficient NRF2 mutant was not altered compared to the wildtype, implying that both p-Ser40 and PKC are dispensable for nuclear NRF2 import since PKC targets exclusively the Ser40 residue in NRF2^16^. It is important to note that the chosen tumor cell lines in this study differ regarding the NRF2-repressor molecule KEAP1. PANC1 cells express the wildtype form of Keap1 that is able to limit NRF2 levels whereas a dysfunctional Keap1 protein in A549 cells provides uncontrolled NRF2 signaling. Therefore, the current data regarding the upstream regulation of NRF2 in A549 cells are not applicable to cancer cells that carry a functional Keap1. Nevertheless, we were able to confirm the previously described dependency of NRF2 on PERK for stabilization via phosphorylation^10^ simply by overexpression of wildtype Keap1 in A549 cells. In consequence, targeting PERK in order to eliminate NRF2 signaling seems only a rational option in tumor cells that carry a non-mutated Keap1 gene.

Moderate amounts of cellular ROS function as important second messenger molecules for proliferation and cell differentiation^20^. Nevertheless, the redox homeostasis is critical for cell survival and depends on adequate expression of antioxidant enzymes, which is mainly regulated by NRF2^21^. NRF2-ARE signaling provides protection from ROS equally for non-malignant tissue and tumor cells, herein triggering chemo-/radioresistance and cancer progression. Therefore, numerous studies have explored the interaction network of NRF2 and discovered many cellular factors and pathways^22^. Regarding a direct participation of NRF2 in DNA damage response (DDR), a previous study, performed under normoxic conditions, found a reduction in RAD51 foci after irradiation when NRF2 was depleted^23^. The authors suggested a transcriptional involvement of NRF2 in the homologous recombination (HR) repair pathway through AREs that they detected upstream of DNA repair genes. However, even though our data confirmed their radiosensitization by NRF2 KD in normoxia, we found this effect much more pronounced in hypoxia and almost completely reversed under reoxygenation. A reasonable explanation is the steady oxygen level of moderate hypoxia (1% O_2_ ≈ 7.5 mmHg) during our experiments, which leads to a maximum of cellular ROS generation^24^ and consequently to a strict hypoxia-related dependency on NRF2. Another work connected NRF2 signaling to cell cycle progression, where p21-triggered NRF2 signaling provided normal cell growth under oxidative stress, thereby avoiding p53-mediated cell death^25,26^. This observation, however, based on results produced in HCT116, COS-1 and MEF cells, does not fit to our data where cell cycle arrest and apoptosis was completely missing after NRF2 decrease. The p53 status of the cell lines cannot explain this discrepancy, since A549 cells express wt *p53* gene but PANC1 cells express an inactive *p53*-mutant (*R273H)*. It is important to mention, that, compared to the common upregulation of the PI3K/AKT pathway during the first 24 h of hypoxia^27,28^, we noticed a NRF2-independent downregulation of activated proliferation factor AKT and cMyc after long-term hypoxic exposure (≥72 h).

The strong proliferation inhibition in our experiments appeared solely in hypoxia and suggested a potential interaction between NRF2 and HIF signaling. Some previous studies reported a synergistic effect of both pathways^29,30^, others showed contrary effects^31,32^. Initially, it was surprising that we could not detect any HIF-1α protein in hypoxia despite upregulated ROS levels after NRF2 KD since increased ROS are known to promote cellular stabilization of HIF-1α^33^. Instead, we noticed a complete dependence of HIF-1α on NRF2 expression, which was also suggested lately by another research group^15^. However, in addition to their finding that NRF2 triggers the transcription of HIF-1α, we detected an interaction on the protein level implying a stabilizing function of NRF2 for HIF-1α. For further clarification, we performed cycloheximide experiments in hypoxia to compare the half-life of HIF-1α with and without NRF2. Owing to its strict dependency on NRF2 though, protein levels of HIF-1α were too low after depletion of NRF2 for a rational analysis of the western blot results (data not shown). Hence it is still elusive whether this protein interaction found between NRF2 and HIF-1α plays a relevant part in the stabilization of HIF-1α.

Interestingly, our gene reporter assays showed a residual HIF activity of roughly 50% after NRF2 depletion when no HIF-1α protein was present, indicating that HIF-2α expression is not influenced by NRF2. Since a recent paper described a compensatory radioprotection by HIF-2α for HIF-1α deficiency^34^, the radioresistance during the presence of NRF2 and HIF-1α relies more likely on NRF2. So far, we cannot definitely say which of the two transcription factor, NRF2 or HIF-1α, is responsible for proliferation under hypoxic conditions although overexpressed HIF-1α failed to rescue cancer growth after NRF2 KD. We cannot exclude either the possibility that HIF-1α is the main determinant but needs NRF2 as a co-transcription factor for efficient expression of its target genes. In line with our negative HIF-1α rescue experiment was a previous study showing that overexpressed HIF-1α triggered cell death under ischemia-like conditions^35^. In contrast, another research group claimed the opposite as they recently found that the growth inhibition induced by NRF2 downregulation could be reversed by exogenous HIF-1α expression in breast cancer cell lines^36^. However, this study seems difficult to interpret since it lacks any methodical information if cells were grown in hypoxia and needs therefore further confirmation.

We performed a computational analysis in more than 450 human cancer cell lines to compare gene expression levels of PERK, NRF2, and HIF-1α. using the online repository “Expression Atlas” (http://www.ebi.ac.uk/gxa/home)^37^: High levels of NRF2 and HIF-1α correlated in the majority of the tested cell lines in contrast to the overall lesser expression of PERK. Notably, in those cell lines expressing high amounts of PERK, we found a correlation together with Hif1a and NRF2 underlining the relevance of the PERK-NRF2-HIF-axis in human cancer. In addition, we used the “Lung Adenocarcinoma (TCGA, Firehose Legacy)” dataset at “http://www.cBioPortal.org”^38^ and found a correlation between KEAP1 mutation and increased expression of various NRF2 target genes (SRXN1, G6PD, GPX2, GCLC, GCLM, and NQO1), which is in line with increased NRF2 activity due to the missing degradation mechanism. Notably, mutated KEAP1 does not correlate with increased expression of HIF1, PERK, and NRF2. However, these findings do not exclude a regulatory mechanism on protein level by direct or indirect interaction as we have shown for HIF1 and NRF2 in this work.

All together, our observations in two radio- and chemoresistant tumor cell lines helped to evaluate limits and chances of each member of the PERK-NRF2-HIF-axis for tumor treatment. The direct targeting of NRF2 might be reasonable to increase radiation sensitivity in oxygen starved tumors by not only inhibiting the antioxidant response but simultaneous disruption of the HIF pathway. On the other hand, the efficiency of targeting NRF2 upstream by inhibiting PERK strictly relies on the presence of functional KEAP1. Phosphorylation of NRF2 by PERK does not affect nuclear translocation or transcriptional activity but exclusively prevents the degradation of NRF2 by KEAP1. The oncogenic KRAS was described as another critical factor for increased NRF2 levels in human cancer^39^. Hence, a combination of PERK and KRAS inhibition might lead to enhanced therapeutic efficacy. KRAS inhibition alone might be a valuable approach for patients with a mutated Keap1 gene since its mechanism of action seems to be KEAP1-independent^40^. Undoubtedly, however, further research effort is necessary to fully understand this complex stress-signaling network in cancer cells.

## Material and methods

### Antibodies and reagents

Antibodies against HO1, NQO1, PARP, c-Myc, P-Akt (S473), PERK, GAPDH, HA, and Flag were from Cell Signaling (Frankfurt/Main, Germany), anti-NRF2 and anti-Laminin-A from Abcam (Cambridge, UK). HIF-1α antibody was purchased from BD (New Yersey, U.S.) and anti-actin antibody was acquired from Sigma-Aldrich (Munich, Germany). HRP-coupled secondary anti-mouse and anti-rabbit antibodies were from Dako (Hamburg, Germany). Chemical compounds were obtained from Sigma (Munich, Germany) except PERK inhibitor GSK2656157, which was from Millipore (Billerica, MA, USA) and dimethyloxaloylglycine (DMOG), which was purchased from Alexis Biochemicals (Loerrach, Germany).

### Cell culture, transfection, and lentiviral production

A549 cells, PANC1 cells, and HEK293T cells were cultured in Dulbecco’s modified Eagle medium (DMEM) High Glucose (Invitrogen, Darmstadt, Germany). The media were supplemented with 10% FBS and 1% penicillin/streptomycin. For normoxic conditions (Nx) cells were incubated with 21% O_2_ and 5% CO_2_. During hypoxic conditions (Hx), cells were kept in a hypoxic chamber (Toepffer Lab System, Göppingen, Germany) with 1% O_2_ and 5% CO_2_. Transient transfection was performed with Viafect (Promega, Mannheim, Germany) as recommended by the manufacturer’s protocol in a ratio of 3: 1 (μl reagent per μg DNA). Lentiviral particles were produced in HEK293T cells as described before^41^. For transduction, 2 × 10^5^ A549/PANC1 cells were incubated with 2 × 10^6^ lentiviral particles together with polybrene (8 μg/μl) over 24 h for enhanced adherence. Transduced cell lines A549-shNRF2, PANC1-shNRF2, and A549-shPERK were selected by puromycin (2 µg/µl) for at least 5 days. By addition of 250 ng/ml doxycycline, gene knockdown was induced for KD experiments.

### Plasmids and short-hairpin RNA (shRNA) sequences

For transient transfections the following plasmids were used: pGL3-8xARE^42^, or pGLHIF1.3^43^, pGL4.74 renilla luciferase (#E6921, Promega), pGFP-NRF2 (pcDNA3-EGFP-C4-Nrf2, #21549, Addgene), Flag-NRF2 (pCDNA3.1 FLAG NRF2, #36971, Addgene), Flag-Keap1 (#28023, Addgene), p(HA)HIF1alpha (P402A,P564A) (#52636, Addgene). To generate plasmid GFP-NRF2-S40A, a modified QuickChange protocol was used^44^ to perform a PCR using pGFP-NRF2 as the template and primers NRF2-S40A-for (5ʹ-GTCGAGAAGTATTTGACTTCGCCCAGCGACGGAAA-GAGTATGAGCTGGAAAA-3ʹ) and NRF2-S40A-rev (5ʹ-GGCGAAGTCAAATAC-TTCTCGACTTACTCCAAGA TCTATATCTTGCCTCCAAAGTATGTCA-3ʹ). pLKO.1-shRNA-NRF2 tet-on (tetracycline inducible) contained the sequence 5ʹ-GCTCCTACTGTGATGTGAAAT-3ʹ of NRF2 mRNA (GenBank acc. no. NM_001145412), pLKO.1-shRNA-PERK tet-on (tetracycline inducible) contained the sequence 5ʹ-GGAACGACCTGAAGCTATAAA-3ʹ of PERK mRNA (GenBank acc. no. NM_004836).

### Sodium dodecyl sulfate polyacrylamide gel electrophoresis (SDS-PAGE) and western blot

After cell lysis in RIPA buffer (50 mM Tris pH 7.5, 2 mM EDTA, 150 mM NaCl, 1% Nonidet P40, 0.1% SDS, 0.5% sodium desoxycholate), including protease/phosphatase inhibitor cocktail (#5872, Cell Signaling), proteins were separated by SDS-PAGE and transferred to polyvinylidene fluoride membrane via Trans-Blot Turbo Blotting System (Bio Rad, California, USA). After blocking the membrane with 5% BSA in TBS-T (50 mM Tris/HCl, 150 mM NaCl, 0.5% Tween-20, pH 7.2) for 1 h, antibody incubation of the membrane was conducted as recommended by the manufacturer. Proteins were detected by using an ECL Kit (#34095, Thermo Fisher Scientific, Oberhausen, Germany) and an FX7 chemoluminescence documentation system (Peqlab, Erlangen, Germany).

### Colony formation assay

To analyze long-term cell survival after exposing cells to increasing doses of radiation, colony formation assay (CFA) was performed: cells were spread out in T25 flasks, KD induced and after incubation for 72 h, cells were trypsinized and seeded in 6-well plates at various densities (400 to 4000 cells per well). During the next 24 h, cells were irradiated with 3 or 5 Gy. Hypoxic cells were kept continuously at an oxygen level of 1% before (16 h), during and after irradiation until the end of the experiment. After 10 days, the grown colonies were fixed with 0.25% paraformaldehyde (PFA) for 20 min, permeabilized with 70% ethanol and dyed with Coomassie Brilliant Blue (0.1 Coomassie blue, 5% acetic acid, 45% methanol) for 1 h. Plates were photographed with the FX7 system using UV-illumination. For analysis colonies of more than 50 cells were counted with ImageJ using a colony area plug-in. Furthermore, Plating efficiency (PE = counted colonies/seeded cells) and survival fraction (SF = colonies formed after treatment/cells seeded × PE) were quantified.

### Luciferase assay

We measured NRF2 transcription activity by performing Luciferase reporter gene assays: 6 × 10^4^ A549 cells were seeded in 24-well plates. Next day cells were co-transfected with 500 ng of pGL3-8xARE (NRF2 activity) or pGLHIF1.3 (HIF activity), together with 100 ng pGL4.74 (renilla luciferase). If required, cells were co-transfected with 500 ng of GFP-NRF2, GFP-NRF2-S40A or various amounts of Flag-Keap1. Transfections were performed using ViaFect (Promega, Madison, U.S.) following the manufacturer’s instructions. Seventy-two hours later, cell lysis was done by using passive lysing buffer (Promega, Madison, U.S.). Firefly and renilla luciferase were measured using the GloMax detection system (Promega) and normalized to renilla values.

### Caspase 3 assay

To quantify levels of apoptosis Caspase-3 activity was measured. Cells were lysated in caspase-3-lysis buffer (Tris (pH 7.3) 50 mM, NaCl 150 mM, Nonidet P40 1%) and protein concentration of the supernatant calculated by using a BCA Kit (Thermo Fisher Scientific). A protein concentration of 20 µg each was prepared with 66 μM DEVD-AMC and 10 mM DTT in caspase substrate buffer and incubated at 37 °C. For 2.5 h, repeated measurements of the released AMC fluorescence were performed every 10 min with a multi-mode microplate reader Synergy^TM^ HT (BioTek, Bad Friedrichshall, Germany). For analysis, bar graphs from the linear range of the reaction were used at time point of 2 h.

### Co-immunoprecipitation

In all, 7 × 10^5^ HEK293T cells were seeded in a 6-well plate and co-transfected with 3 µg plasmid of Flag-NRF2 and 3 µg of plasmid p(HA)HIF1alpha-P402A-P564A. After 48 h of incubation, cells were lysed in caspase lysis buffer and incubated for 4 h at 4 °C with Flag antibody. As a control IP, lysate without antibody was used. Afterwards, the lysate was incubated for 1 h with protein S and G magnetic beads (Cell Signaling Technologies, Danvers, USA). Beads were washed 4x in caspase lysis buffer and finally transferred to SDS-sample buffer containing DTT and were heated at 99 °C for 10 min before loading on a 7.5% polyacrylamide gel.

### Fluorescence microscopy

For localization experiments 3 × 10^4^ cells were plated on glass cover slides in a 24-well plate and subsequently the KD was induced. Next day cells were transfected with 500–1000 ng of plasmids pEGFP-NRF2 or pEGFP-NRF2-S40A. After 48 h, Thapsigargin (1 µM), Tunicamycin (1 µM), or PERK-inhibitor GSK2656157 (10 µM) were added. Twenty-four hours later, the medium was removed and the cells were fixed with 0.4% PFA in PBS and counterstained with 3.3 μg/ml HOECHST32444. Visualization was done with a Zeiss LSM510 inverse confocal microscope with a 63×/1.2 NA oil immersion lens (Carl Zeiss, Heidelberg, Germany).

### CellROX

Oxidative stress measurements were evaluated using CellROX Green Reagent (Thermo Fischer). In all, 6 × 10^4^ PANC1 cells or 4 × 10^4^ A549 cells have been spread out and subsequently KD was induced. After 3 days under normoxic or hypoxic conditions (1%O_2_), cells were incubated with 5 µM CellROX for 2 h. The CellROX itself is non-fluorescent, but if it gets oxidized in cells, the excitation at 488 nm leads to the emission of intense green fluorescence with wavelengths around 520 nm. The fluorescence was detected using an Eclipse Ts2-FL fluorescence microscope with a 20x CFI LWD objective (Nikon, Amsterdam, Netherlands) and quantified with the image processing and analysis software IMAGEJ.

### BrdU cell proliferation assay

Cell proliferation was assessed using the BrdU Cell Proliferation ELISA Kit (Ab126556, Abcam) following the manufacturer’s instructions. Briefly, 6 × 10^3^ cells were plated in a 96-well plate and subsequently the KD was induced. After incubation for 72 h in hypoxia, BrdU was added to the cells for 3 h. Subsequently, cells were subjected to fixation, permeabilization, and DNA denaturation. Next, cells were incubated with anti-BrdU antibody for 1 h, washed and incubated with peroxidase-conjugated secondary antibody. Finally, the colored reaction that indicates cell proliferation was quantified using a spectrophotometer at a wavelength of 450 nm.

### Cell cycle analysis

For cell cycle analysis, 4 × 10^5^ cells were lysed following 96 h after NRF2 KD in 350 μl hypotonic citrate buffer (0.1% sodium citrate, 0.1% Triton X-100) containing 50 μg/ml PI for 30 min. Subsequently, intact single nuclei (as discriminated by PE-area versus PE-width gating) were analyzed for DNA content by FACS. At least 10,000 nuclei per sample were analyzed using cell analyzer FACSCanto^TM^ II system (BD Bioscience, Heidelberg, Germany).

### Statistical analysis

All experimental results were confirmed in at least three independent experiments. In bar graphs with bars not less than three independent samples were taken. Bars represent the mean of samples plus standard deviation (SD) of one representative experiment. For multiple group comparison, two-way ANOVA was used, followed by Tukey post-hoc test using Prism software (GraphPad Software, Inc., La Jolla, CA, USA). In all figures, n.s. indicates *p* > 0.05, *indicates *p* < 0.05, **indicates *p* < 0.01, ***indicates *p* < 0.001, and ****indicates *p* < 0.0001.

## Data Availability

The original data that support the findings of this study are available from the corresponding author upon reasonable request.
